# Intraoperative neurophysiological monitoring in pediatric vascular neurosurgery: a review of the literature and institutional case series

**DOI:** 10.1007/s00381-026-07228-6

**Published:** 2026-03-25

**Authors:** Marco Galeazzi, Nicole Montereale, Michele Di Domenico, Federico Bianchi, Paolo Frassanito, Luca Massimi, Gianpiero Tamburrini

**Affiliations:** 1 Department of Neurosurgery, Fondazione Policlinico Universitario Agostino Gemelli IRCCS, Rome, Italy; 2 Pediatric Neurosurgery, Fondazione Policlinico Universitario Agostino Gemelli IRCCS, Rome, Italy

**Keywords:** Arteriovenous malformation, Cavernoma, Neuromonitoring, Awake surgery

## Abstract

**Purpose:**

Intraoperative neurophysiological monitoring (IONM) has become an essential tool in neurosurgery, yet its application in pediatric cerebrovascular pathology remains scarcely documented. This paper aims to evaluate current evidence regarding the use of IONM in pediatric vascular neurosurgery and to present an institutional experience with AVM and cavernoma surgery performed with intraoperative neuromonitoring.

**Methods:**

A scoping review of literature was conducted and a 13-year retrospective pediatric institutional case series was reported, including 8 AVMs and 4 cavernomas operated with IONM.

**Results:**

Only five studies met the inclusion criteria, mostly consisting of case reports or small series, confirming the paucity of literature in this field. Reported experiences consistently described IONM as useful for detecting ischemia, guiding subcortical dissection, identifying safe brainstem or cortical entry zones, and supporting functional preservation. In the institutional series, IONM enhanced surgical resection safety by guiding temporary clipping in AVMs, facilitating identification of eloquent pathways during nidus dissection, and assisting in cortico-subcortical mapping in cavernoma surgery.

**Conclusions:**

Despite limited published evidence, our institutional experience supports the value of IONM as a technically feasible and potentially useful adjunct in pediatric vascular neurosurgery. For these malformations characterized by a high degree of surgical complexity, intraoperative neuromonitoring may further expand the armamentarium available to surgeons.

**Supplementary Information:**

The online version contains supplementary material available at 10.1007/s00381-026-07228-6.

## Introduction

Intraoperative neurophysiological monitoring (IONM) has gained a key role in neurosurgery to merge radical surgical treatment with maximum patient safety.

IONM is being increasingly employed in both adult and pediatric patients; however, certain technical aspects require adaptation for use in children [4] and controversies regarding its indications persist [17].

Notably, there is a paucity of literature addressing the use of intraoperative monitoring in pediatric vascular neurosurgery.

This paper integrates a scoping literature review and a retrospective institutional case series to evaluate the use and feasibility of IONM in the most frequent pediatric cerebrovascular malformations: arteriovenous malformations (AVMs) and cavernomas.

## Historical background

A search of electronic databases (PubMed, Scopus) was performed using the following search terms: (intraoperative neuromonitoring OR IONM OR intraoperative neurophysiological monitoring) AND (pediatric neurosurgery). The latest search was conducted in October 2025.

This review was conducted in accordance with established guidelines, including the PRISMA (Preferred Reporting Items for Systematic Reviews and Meta-Analyses) [13] framework, to ensure a rigorous identification and critical evaluation of relevant studies.

Studies were included if they reported the use of IONM in pediatric cerebrovascular neurosurgery.

Exclusion criteria included studies published in languages other than English, studies on the use of IONM in surgery for spine pathologies, brain tumors, epilepsy, or Chiari malformation.

For each included study, the following data were extracted: first author, publication year, number of patients, underlying pathology, surgical approach, and type of IONM.

Data were summarized descriptively. Tables were prepared to present the type of studies, number of patients, IONM methodology, limitations, and conclusions.

A total of 90 articles were screened after duplicate removal, with 5 studies meeting inclusion criteria. Main features of included studies are reported in Table 1.

**Table 1:** Summary of studies included in the systematic review

Author	Type of study	No. of patients	Type of IONM	Limitations	Conclusions
López [9]	Retrospective case series	121 AVMs	SSEPs, BAEPs, MEPs, EEG, and EMG	Study published more than 15 years ago and based on a period between 1995 and 2004 No clear data on precise number of operated versus embolized AVMs. No data on single cases and outcomes	IONM is a safe and useful tool in the intraoperative management of pediatric cerebrovascular diseases. It allows early detection of cerebral ischemia which can influence perioperative decisions
Roth [16]	Retrospective case series	2 (1 AVM; 1 cavernoma)	MEPs, SSEPs, subcortical mapping using CUSA	Small cohort. In subcortical stimulation, relationship of 1 mA per 1 mm has still to be proven in children	CUSA-based subcortical mapping enables an efficient and uninterrupted surgical workflow, increasing patient safety
Roth [17]	Retrospective case series	3 (pathology not specified)	MEPs, SSEPs, cortical and subcortical mapping	Small cohort No specific data regarding the type of vascular pathology, outcomes, or patient characteristics Risk of population’s overlap with the previous study	IONM may play a role in pediatric vascular neurosurgery, especially for lesions in proximity to the CST and motor area
Yeole [24]	Case report	1 (cavernoma)	Motor cortex mapping using a NIM© nerve monitoring system during awake surgery	The use of this system does not allow other modalities of IONM	The authors described this technique as an effective alternative for surface mapping in case of absence of unavailability of standard IONM modalities
Ndandja [11]	Case report	1 (cavernoma)	Brainstem-evoked potentials, motor mapping	Lack of details in the description of the monitoring technique used	IONM reported as useful tool in identifying entry points, guiding safe resection and manipulation with minimal risk of damage to surrounding neural tissue
Present series	Retrospective case series	12 (8 AVMs, 4 cavernomas)	MEPs, SSEPs, VEPs, cortical and Subcortical mapping, EEG	Small cohort with heterogeneity in lesion location, follow-up, and imaging timing	IONM provides real-time assessment on functional pathway integrity that no other modality can currently offer

*AVM* Arteriovenous malformation, *BAEPs* Brainstem auditory evoked potentials, *CST* Corticospinal tract, *CUSA* Cavitron UltraSonic Aspirator, *EEG* Electroencephalography, *EMG* Electromyography, *IONM* Intraoperative neurophysiological monitoring, *MEPs* Motor evoked potentials, *MRI* Magnetic resonance imaging, *NIM©* Nerve Integrity Monitoring system (Medtronic NIM® Response 4.0), *SSEPs* Somatosensory evoked potentials

### Literature review

IONM in adult neurosurgery has established itself in various fields, including vascular pathology [14, 21]. Conversely, very few studies address the use of IONM in pediatric vascular surgery.

However, the limited data available in the literature seem to support various applications of IONM in this field.

Despite technical difficulties, IONM demonstrates validity and clinical utility in the pediatric population as in adults [17] and was described as an efficient method in detecting signs of cerebral ischemia, guiding perioperative decisions [9].

One of the most commonly reported applications is subcortical mapping, particularly helpful in allowing continuous assessment during the active stages of lesion removal [16, 24].

Simultaneously, monitoring of motor function performed throughout the procedure is a complementary approach to detect a worsening and guiding the resection, enhancing patient safety [17]. This strategy is essential in high-risk procedures, like brainstem cavernomas, for precise resection of the lesion with minimal damage to the surrounding neural tissue [11].

## Clinical presentation

AVMs are high-flow cerebrovascular lesions consisting of feeding arteries, draining veins, and a dysplastic nidus composed of a tangle of abnormal vessels.

Pediatric brain arteriovenous malformations are an uncommon condition but are characterized by a significant clinical burden and complexity of management.

Although rare in pediatric patients, with an estimated prevalence ranging from 0.014 to 0.028% [5], they are responsible for approximately half of all intracranial hemorrhages in this population [3, 10].

Compared with adult cases, pediatric AVMs demonstrate a more aggressive clinical course, characterized by a higher recurrence rate [7], increased annual risk of hemorrhage [5], and a higher probability of presenting with rupture [8, 10, 12].

In addition to hemorrhagic onset, other common presentations include seizures (up to 30% of cases), headaches, focal neurological symptoms, and, in neonates, congestive heart failure [23].

Approximately 15% of lesions are incidental findings [12].

Another major cause of spontaneous intracranial hemorrhage in pediatric age is cerebral cavernous malformations (CCMs). One quarter of cavernomas affects children [19] and their natural history seems to have a more aggressive course than in adults, with higher hemorrhage risk and symptomatic presentation [2, 22].

Hemorrhagic presentation due to extralesional bleeding leads to acute expansion and mass effects on adjacent brain tissue [15].

Intralesional bleeding is associated with a slowly dimensional increase that might be responsible for other clinical presentations such as headache or focal neurological deficits, while seizures can result from both subacute bleeding and chronic inflammatory changes around the lesion [15].

Twenty-five to 45% of pediatric patients with CCMs are asymptomatic and discovered incidentally [6].

## Diagnosis

The acute onset of neurological symptoms in a child often leads to a brain CT scan which, in the case of hemorrhagic presentation of an AVM or cavernoma, reveals the presence of intraparenchymal hemorrhage.

In the presence of an underlying AVM, completion with CT angiography is usually able to detect the presence of a nidus with the typical vascular alterations associated with this malformation. Then, a cerebral angiography is usually performed as the gold standard for the study of the malformation’s angioarchitecture.

In the case of hemorrhage sustained by a cavernoma, MRI is the diagnostic examination of choice, as these malformations are angiographically silent, while MRI is able to detect an underlying CCM.

In non-acutely hemorrhagic lesions, advancements in MRI technologies like susceptibility-weighted imaging (SWI) have led to the increased detection of incidental CCMs in the pediatric population [15].

## Management, prognosis, and outcomes

### IONM in pediatric vascular neurosurgery

Acute bleeding can be a life-threatening condition, requiring emergency surgical treatment.

Unruptured AVMs or cavernomas might also require surgery when symptoms related to these malformations develop and persist or if they cause seizures refractory to medical treatment.

These lesions can be placed in locations where both bleeding and surgical treatment might have particularly severe consequences.

IONM is among the major innovations in neurosurgery since it assesses the integrity of neural pathways during surgery, preventing new-onset neurological deficits.

Given the high complexity of these pathologies, along with their considerable morbidity and mortality, it is essential to establish an optimal treatment strategy aimed at achieving their complete and permanent resection while minimizing treatment-related risks [20]. IONM has established itself as a solid answer to this demand.

IONM includes two categories of techniques: monitoring and mapping.

Monitoring techniques—such as MEPs, SSEPs, VEPs, and cortico-bulbar evoked potentials—allow continuous assessment of a specific pathway throughout the surgical procedure.

Mapping techniques provide punctual information about a specific structure at a specific moment.

In supratentorial surgery, direct stimulation can be used to identify the primary motor cortex, subcortical tracts, language areas, and oculomotor nerves.

Language mapping is possible only during awake surgery and is considered a form of “negative mapping,” meaning that the patient performs tasks, and interruption of the task during stimulation indicates involvement of a language-related area.

Phase reversal is another example of supratentorial mapping techniques: It consists of recording a SSEP directly from the cerebral cortex using strip electrodes. If sensory responses are recorded from the motor cortex, their polarity will be inverted. Identifying the point at which this inversion occurs allows localization of the central sulcus.

**Fig. 1:**
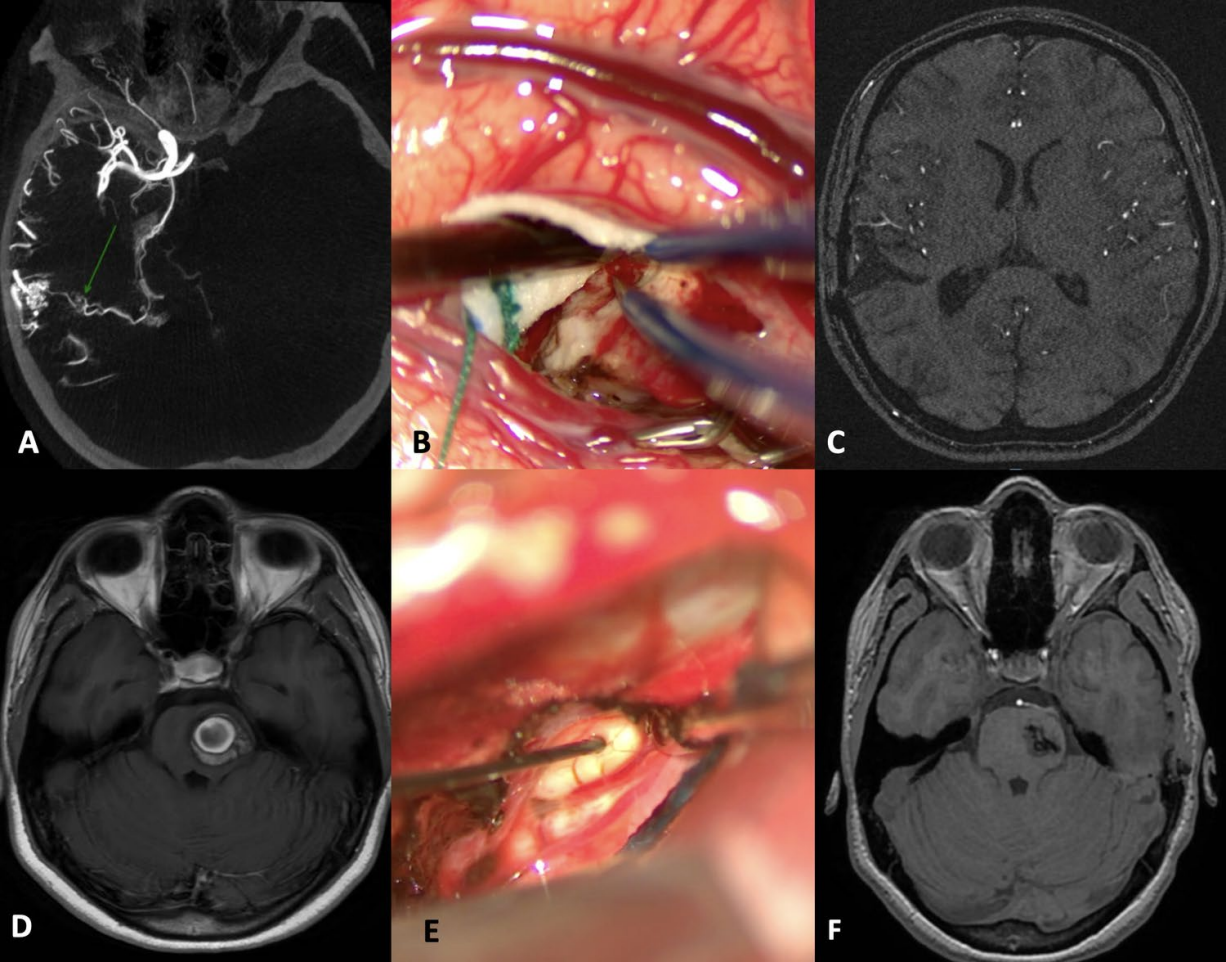
**A** A 16-year-old patient (*AVM case number 4*) presenting with intraparenchymal hematoma in the right temporal lobe sustained by a compact-nidus AVM fed by distal branches of the right MCA and draining into the ipsilateral Labbé vein. **B** The patient underwent surgery with MEP and SSEP monitoring and the use of a subcortical stimulator. The nidus was circumscribed with the aid of a stimulated aspirator (no response evoked with stimulation up to 12 mA) and by coagulating the comb-like branches until the feeder originating from the middle cerebral artery was identified at the base. **C** At the 2-year follow-up, complete excision of the malformation is confirmed and clinically the patient presents no neurological deficits. **D** A 17-year-old patient (*cavernoma case number 2*) was diagnosed with a cavernous malformation in the left pons after an episode of severe headache. An MRI at 3 months showed an increase in the size of the lesion with signs of recent bleeding. She therefore underwent surgery through a left subtemporal approach. **E** Mapping of the midbrain surface was performed to identify the entry zone without sensory-motor responses. The dissection was performed with simultaneous mapping and neurophysiological monitoring to ensure safe excision of the malformation. **F** A follow-up MRI scan at 6 months documented complete removal of the cavernoma and the patient showed an improvement of the hyposthenia presented after surgery, with independent walking possible

In infratentorial surgery, mapping techniques are used to identify motor nuclei on the floor of the fourth ventricle and motor cranial nerves (Fig. 1).

The use of IONM in pediatric patients represents a significant challenge for several reasons.

In children, the nervous system is less developed than in adults, making the elicitation of evoked potentials more difficult even in the absence of evident clinical deficits, and activation thresholds tend to be higher.

Moreover, in supratentorial surgery, the application of high-intensity stimuli can activate the subcortical white matter, bypassing the lesion site and increasing the risk of false negatives.

The small size of the skull, combined with the space occupied by the surgical field, often prevents correct electrode placement and, especially in very young children, corkscrew electrodes cannot be used. In these cases, subcutaneous needle electrodes may be applied but significantly less stably.

Peripheral electrode placement also differs from adults: The available surface area is smaller, and in more complex monitoring procedures (such as monitoring in infratentorial surgery), proper setup becomes more challenging.

All IONM techniques are strongly influenced by the type of anesthesia used, and this interaction is even more complex in children due to factors such as immaturity of the motor cortex and subcortical motor pathways and higher activation thresholds [18]. To minimize the effect of anesthetic drugs on the reliability of recordings, total intravenous anesthesia (TIVA) is preferred in our institution, and neuromuscular blocking agents are usually avoided or limited to minimal doses during induction and intubation only. Hypothermia and variations in arterial blood pressure can also induce changes in evoked potentials; therefore, it is essential to monitor and maintain these parameters within stable ranges.

## Exemplary case description

With our institutional case series, we aim to present our experience in the use of neurophysiological monitoring in pediatric vascular pathology.

The institutional case series is reported in accordance with the PROCESS guidelines [1].

This case series is intended as a descriptive feasibility experience rather than an outcomes study, given the limited case volume and heterogeneity. No causal inference regarding the impact of IONM on outcomes was intended, and no comparative analysis with non-monitored cases was performed.

Surgical reports and discharge letters from the last 13 years were analyzed and 8 cases of brain AVMs and 4 cavernous malformations operated on with the use of IONM were identified.

Demographics, clinical history, imaging findings, surgical details, IONM reports, post-operative complications, and follow-up data of these patients were collected retrospectively.

At our facility, IONM is performed by a technician physically present throughout the entire surgical procedure. In cases requiring complex interpretation or where mapping/testing procedures are necessary, a neurosurgeon specialized in neurophysiology is called to the operating room to provide on-site supervision. The baseline electrophysiology acquisition is obtained after the anesthesiologic procedures and patient positioning for surgery due to difficulty in achieving cooperation for high-quality pre-operative electrophysiological recordings and inter-individual variability in pharmacological responses.

All procedures were performed in accordance with the principles outlined in the Declaration of Helsinki. Parents signed informed consent regarding procedures, data collection, and use.

Ethical approval was waived by the Ethics Committee of Catholic University of Sacred Heart in view of the retrospective nature of the study, and all the procedures being performed were part of the routine care.

The authors did not receive support from any organization for the submitted work and have no competing interests to declare that are relevant to the content of this article.

Patients’ features are reported in Tables 2 and 3, while Tables 4 and 5 show the details of IONM’s use and clinical outcomes.

**Table 2:** Clinical features of patients surgically treated with IONM for AVM

Patient	Age	Sex	Onset	Symptoms	Grade (SM/LY)	Urgent surgery*	Other treatments	AVM resection (total/incomplete)	Complications	Residual at FUP
1	9	M	Ru	Headache, left hemiparesis	2/2	N	Pre-operative embolization	Total	No	No (3 years)
2	6	M	Ru	Seizure	4/2	Y	Previous hematoma evacuation and partial excision	Total	No	No (3 years)
3	4	M	Ru	Headache, vomiting	2/1	Y	Previous hematoma evacuation	Total	No	Micro-residual (3 years)
4	16	M	Ru	Headache, vomiting	1/1	Y	No	Total	No	No (4 years)
5	17	F	Ru	Headache	1/1	Y	No	Total	No	No (1 year)
6	11	M	Ru	Seizure	2/2	Y	Previous embolization and decompressive craniectomy	Total	Post-embolization bleeding and ischemia	No (6 months)
7	8	M	Ru	Headache, neurological deterioration	2/2	Y	Previous hematoma evacuation and partial excision	Total	No	No (immediate post-operative imaging)
8	13	F	Ru	Headache, vomiting	1/1	Y	No	Total	No	No (immediate post-operative imaging)

*“Urgent surgery” indicates whether an urgent surgery for hematoma evacuation was performed prior to elective surgery for resection of the malformation

*AVM* arteriovenous malformation, *F* female, *FUP* follow-up, *LY* Lawton–Young, *M* male, *N* no, *Ru* rupture, *SM* Spetzler–Martin, *Y* yes

**Table 3:** Clinical features of patients surgically treated with IONM for cavernoma

Patient	Age	Sex	Onset	Symptoms	Location	Urgent surgery*	Total surgeries	Resection (total/incomplete)	Complications	Residual at final FUP (years)
1	14	F	Seizure	Right hemiparesis	Left Rolandic	No	1	Total	No	No (5 years)
2	17	F	Headache	Headache	Pons	No	1	Total	No	No (1 year)
3	17	F	Seizure	None	Left temporal	No	1	Total	No	No (4 years)
4	16	M	FUP for familial cavernomatosis	Left facial hypoesthesia	Pons	No	3	1 st and 2nd surgery: incomplete 3rd: complete	No	No (8 years)

*Urgent surgery indicates whether an urgent surgery for hematoma evacuation was performed prior to elective surgery for resection of the malformation

*F* female, *FUP* follow-up, *M* male

**Table 4:** Intraoperative neurophysiological monitoring details and clinical outcomes in the pediatric AVM cohort

Patient	Location	Type of IONM	IONM pre	IONM post	Intraoperative IONM utility	mRS pre	mRS at FUP
1	L frontal	MEPs + SSEPs	Present	Unchanged	Neuromonitoring	3	0
2	R temporal + R occipital	MEPs + SSEPs + VEPs, EEG	SEPs/MEPs: left arm and leg absent VEPs: left reduction	Unchanged	Neuromonitoring + temporary clipping evaluation	4	2
3	R temporal	MEPs + SSEPs, EEG	MEPs present SEP: lower limbs not evocable	Unchanged	Neuromonitoring	2	0
4	R temporal	Mapping MEPs + SSEPs Subcortical stimulation EEG	Present No activations at 12 mA subcortical stimulations	Unchanged	Neuromonitoring + subcortical mapping during nidus dissection	1	0
5	R temporal	MEPs + SSEPs EEG	Present	Unchanged	Neuromonitoring	1	0
6	L frontal	Mapping MEPs + SSEPs EEG	Present	50% upper limb SSEP reduction 50% upper limb MEP reduction	Neuromonitoring	4	2
7	R parietal	MEPs + SSEPs	Present	Unchanged	Neuromonitoring	3	1
8	R cerebellar hemisphere	MEPs + SSEPs	Present	Unchanged	Neuromonitoring	2	1

*EEG* electroencephalography, *F* female, *FUP* follow-up, *L* left, *M* male, *R* right, *MEPs* motor evoked potentials, *SSEPs* somatosensory evoked potentials, *IONM* intraoperative neurophysiological monitoring, *VEPs* visual evoked potential

**Table 5:** Intraoperative neurophysiological monitoring details and clinical outcomes in the pediatric cavernoma cohort

Patient	Location	Type of IONM	IONM pre	IONM post	Intraoperative IONM utility	mRS pre	mRS at FUP
1	Right frontal	SSEPs, MEPs, phase reversal, subcortical stimulation, EEG	MEPs: upper limb absent	Unchanged	M1 identification, subcortical stimulation during resection	3	2
2	Pons	SSEPs, MEPs, corticobulbar III and IV, direct nerve stimulation, subcortical stimulation, EEG	Present	Unchanged	Individuation of safe entry point, neuromonitoring	1	2
3	Left temporal	Awake surgery, MEPs, SSEPs, mapping	Present	Unchanged	Speech arrest identification, neuromonitoring	0	0
4	Pons	MEPs, SSEPs, mapping	Present	Unchanged	IV ventricle floor mapping, facial colliculus identification; neuromonitoring	0	3

*EEG* electroencephalography, *F* female, *FUP* follow-up, *IONM* intraoperative neurophysiological monitoring, *L* left, *M1* primary motor area, *M* male, *MEPs* motor evoked potentials, *mRS* Modified Rankin Scale, *R* right, *SSEPs* somatosensory evoked potentials, *VEPs* visual evoked potential

### Illustrative cases

#### AVM case number 2 (Fig. 2)

**Fig. 2:**
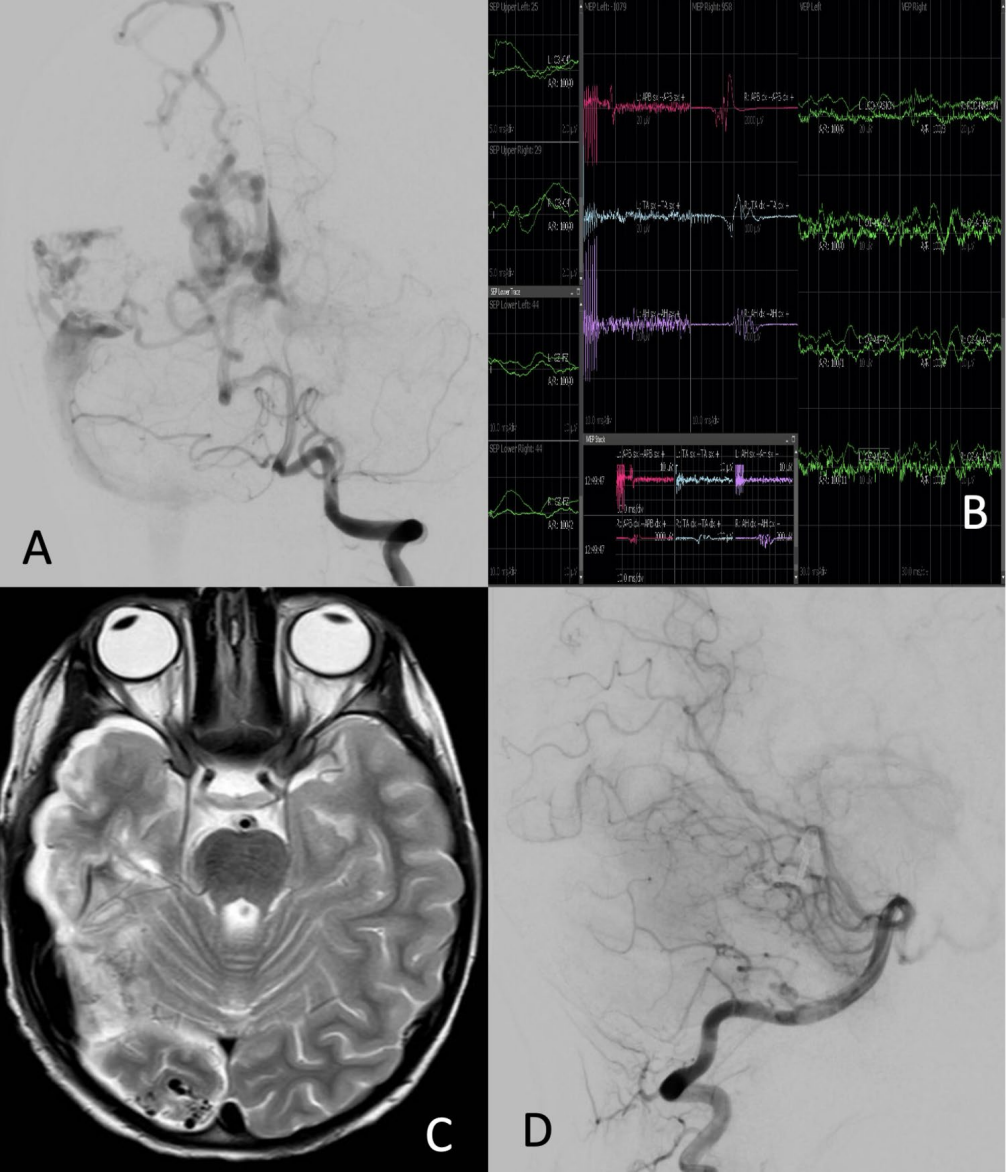
AVM case number 2. **A** Cerebral angiography documented the presence of two cerebral AVMs in the superficial occipital and temporal region. The occipital AVM was fed by an occipital branch of the right PCA and by a posterior temporo-occipital branch of the ipsilateral MCA. Two venous collectors were directed to the right internal cerebral vein and the superior sagittal sinus. The temporal AVM appeared dispersed into three small nidal compartments as a result of the previous surgery, fed by a temporal branch of the PCA and the posterior temporal branch of the MCA, with drainage to the transverse sinus and superior sagittal sinus. **B** MEP, SSEP, and VEP monitoring: before surgery, left SSEPs and MEPs were absent, and left VEPs were also reduced. At the end of the procedure, potentials were unchanged from baseline. **C** Brain MRI before the last surgery for resection of the occipital AVM. **D** Post-operative angiography at 3 years showing no residual malformation

A 6-year-old patient presented with a generalized critical episode. CT angiography showed a right temporo-parietal hematoma associated with two AVMs. The patient underwent urgent surgery at another center for evacuation of the hematoma, partial resection of the temporal malformation, and clipping of the flow-related MCA aneurysm (presumed cause of bleeding).

The patient was transferred to our center where physical examination revealed left homonymous hemianopsia and left hemiparesis, and cerebral angiography was performed*.*

One month later, the patient underwent surgery to remove the right temporal malformation with MEP, SSEP, and VEP monitoring.

During the operation, the assessment of the neurophysiological status, which remained unchanged from baseline, was particularly helpful during the temporary clipping of the feeders coming from the MCA.

One month later, endovascular occlusion of the feeders coming from the MCA was performed using acrylic glue, and 1 week later, surgery was performed to completely remove the occipital malformation.

At the 3-year follow-up, the patient presented with mild left hemiparesis, walking independently with minimal uncertainty and persistent left homonymous hemianopsia*.*

Radiological examinations showed no residual malformation.

#### Cavernoma case number 3 (Fig. 3)

**Fig. 3:**
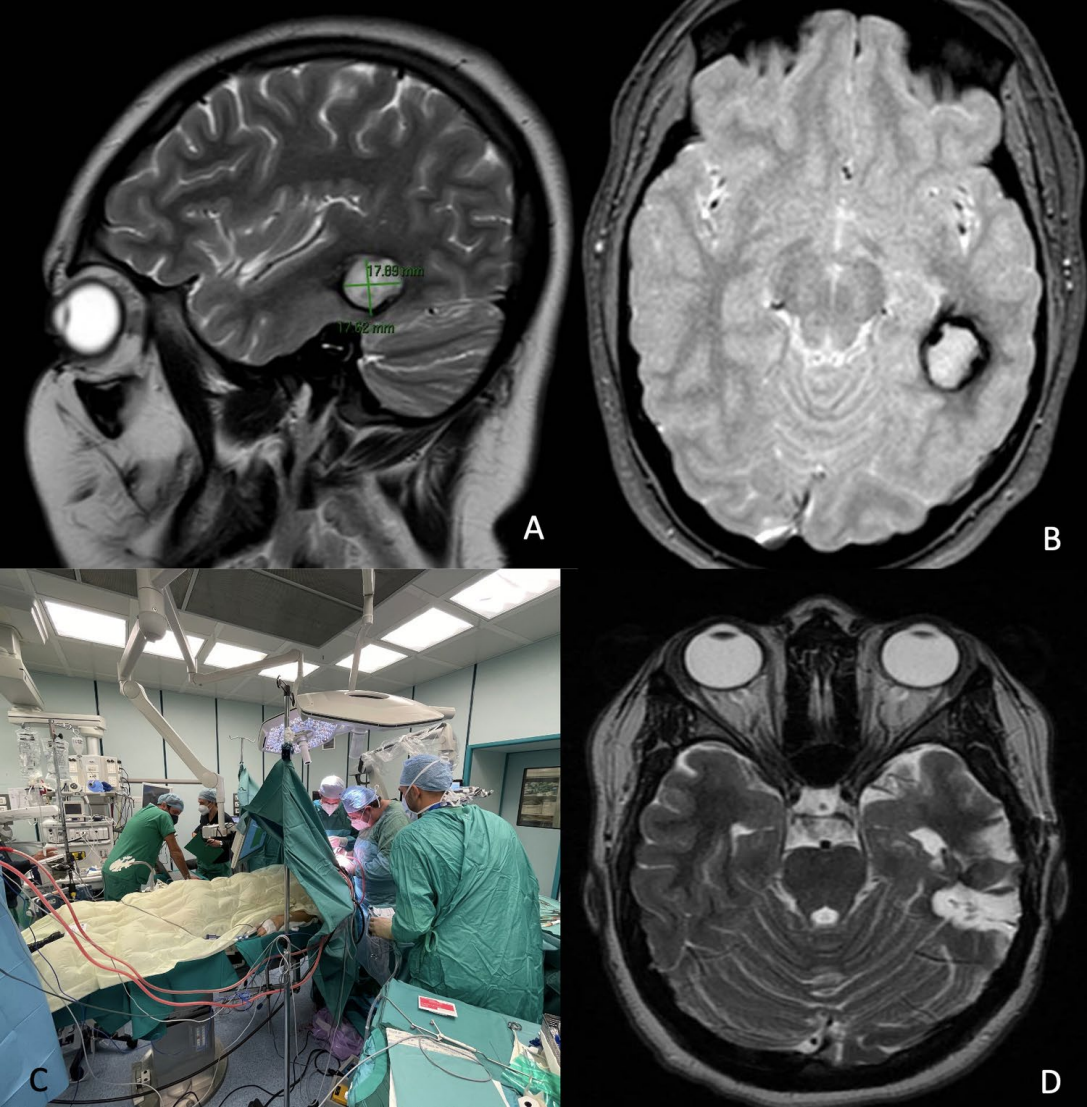
Cavernoma case number 3. **A**, **B** Pre-operative brain MRI showing the cavernoma in the left posterior temporal lobe with an adjacent developmental venous anomaly. **C** Intraoperative setting for awake surgery with collaboration of neurosurgeons, neurologists, and IONM team. **D** Post-operative MRI at 4 years showing complete excision of the malformation

A 17-year-old patient whose recent medical history began with headache, slurred speech, and nausea. The patient underwent a CT scan and an MRI of the brain, which documented the presence of a left posterior temporal cavernoma with signs of recent bleeding.

Five months later, the patient underwent awake surgery. Cortical mapping was performed to identify Wernicke’s area by recording typical signs like occurrence of counting errors. Subcortical mapping guided the preservation of language function detecting the onset of semantic paraphasias, particularly in the superomedial side of the dissection.

The final MRI showed complete resection of the malformation, and the patient remained neurologically intact.

## Discussion

In the context of AVMs, IONM provided helpful intraoperative feedback during critical stages of dissection. Particularly, it guided deep dissection phases of the resection and integrated profitably with the execution of temporary clipping of arterial feeders, achieving favorable modifications of the AVM’s intraoperative hemodynamics without causing damage to healthy parenchyma.

Comparable findings were observed in the treatment of cavernous malformations, in which IONM allowed the identification of safe entry points (particularly for brainstem cavernomas or near eloquent cortical areas) and in subcortically mapping neural tracts during the malformation dissection.

The usefulness of awake surgery should also be highlighted, representing the highest expression of reconciling radical resection with functional preservation.

Depending on the surgical location and the structures involved, different IONM techniques can be combined to reduce the risk of permanent neurological deficits. Despite the additional challenges associated with pediatric monitoring, the potential benefit is considerable: Intraoperative feedback on actual or impending neural injury can modify the surgical strategy in real time, and we believe that it can be valuable for preserving the integrity of the neural pathways under investigation.

Vascular neurosurgery encompasses heterogeneous conditions, each requiring several surgical strategies and, consequently, different intraoperative neurophysiological monitoring techniques.

The approach in cavernoma surgery is almost similar to that of intra-axial tumor lesions, in which IONM plays an “anatomical” role in identifying eloquent areas and entry points and guiding dissection in areas in contact with speech or motor zones.

In AVM surgery, in addition to this role, which is present in the case of nidus adjacent or located in eloquent areas, IONM also plays a role in preserving the integrity of normal blood flow to healthy parenchyma.

AVM surgery is classically composed of phases of exposure of the malformation, pial and parenchymal dissection, and finally resection.

In the first steps, IONM might facilitate the understanding of the AVM’s angioarchitecture, allowing distinction among transit, bystander, or feeding arteries.

During dissection, neurophysiological monitoring effectively integrates with temporary clipping, signaling early damage due to a vascular occlusion detecting a reversible loss of motor evoked potentials following the clipping of an en-passage artery. In these phases, IONM provides essential information in the decision-making process during dissection, clipping, or vessel sacrifice.

Although intraoperative ultrasound and neuronavigation systems provide information on the anatomical location of eloquent areas, monitoring remains the only tool capable of assessing their functional integrity and mapping is the only technique capable of providing certain information about the eloquence of an anatomical structure.

## Limitations

This study presents several limitations. First, the institutional case series includes a small number of heterogeneous cases treated over a long time span, limiting statistical power and generalizability. Second, no control cohort was available for comparison, and therefore, no conclusions regarding outcome superiority can be drawn. Third, variability in lesion type, anatomical location, and clinical status introduces confounding.

## Conclusions

The evidence available on the use of IONM in pediatric vascular neurosurgery remains extremely limited, with only a small number of case reports and small case series published.

We presented our institutional experience with the aim of emphasizing how IONM represents a technically feasible tool that may assist the surgical treatment of pediatric cerebrovascular malformations. Although technical challenges are more prominent in children, IONM can provide real-time assessment on functional pathway integrity that no other modality can currently offer.

In the setting of pediatric vascular neurosurgery, an injury may occur either from direct damage to relevant structures or alteration in vascular flow; therefore, IONM should be considered in the surgeon’s armamentarium to provide the safest and most effective treatment to patients.

## Supplementary Information

Below is the link to the electronic supplementary material.

ESM 1 JPEG (190 KB)

## Data Availability

No datasets were generated or analysed during the current study.

## Abbreviations

AVM: Arteriovenous malformation
BAEPs: Brainstem auditory evoked potentials
CCM: Cerebral cavernous malformation
CNS: Central nervous system
CST: Corticospinal tract
CT: Computed tomography
CTA: Computed tomography angiography
CUSA: Cavitron UltraSonic Aspirator
EEG: Electroencephalography
EMG: Electromyography
EPs: Evoked potentials
FUP: Follow-up
IONM: Intraoperative neurophysiological monitoring
LY: Lawton–Young
M1: Primary motor cortex
MCA: Middle cerebral artery
MEP: Motor evoked potential
MRI: Magnetic resonance imaging
mRS: Modified Rankin Scale
NIM®: Nerve Integrity Monitoring
PCA: Posterior cerebral artery
PRISMA: Preferred Reporting Items for Systematic Reviews and Meta-Analyses
PROCESS: Preferred Reporting Of Case Series in Surgery
VEP: Visual evoked potential
SM: Spetzler–Martin
SSEP: Somatosensory evoked potential
SWI: Susceptibility-weighted imaging
TIVA: Total intravenous anesthesia
