# Supplementary figures and images for: Intraoperative neurophysiological monitoring in pediatric vascular neurosurgery: a review of the literature and institutional case series
Source: Childs Nerv Syst. 2026 Mar 25;42(1):131. doi: 10.1007/s00381-026-07228-6 (PMC13013254; doi:10.1007/s00381-026-07228-6)

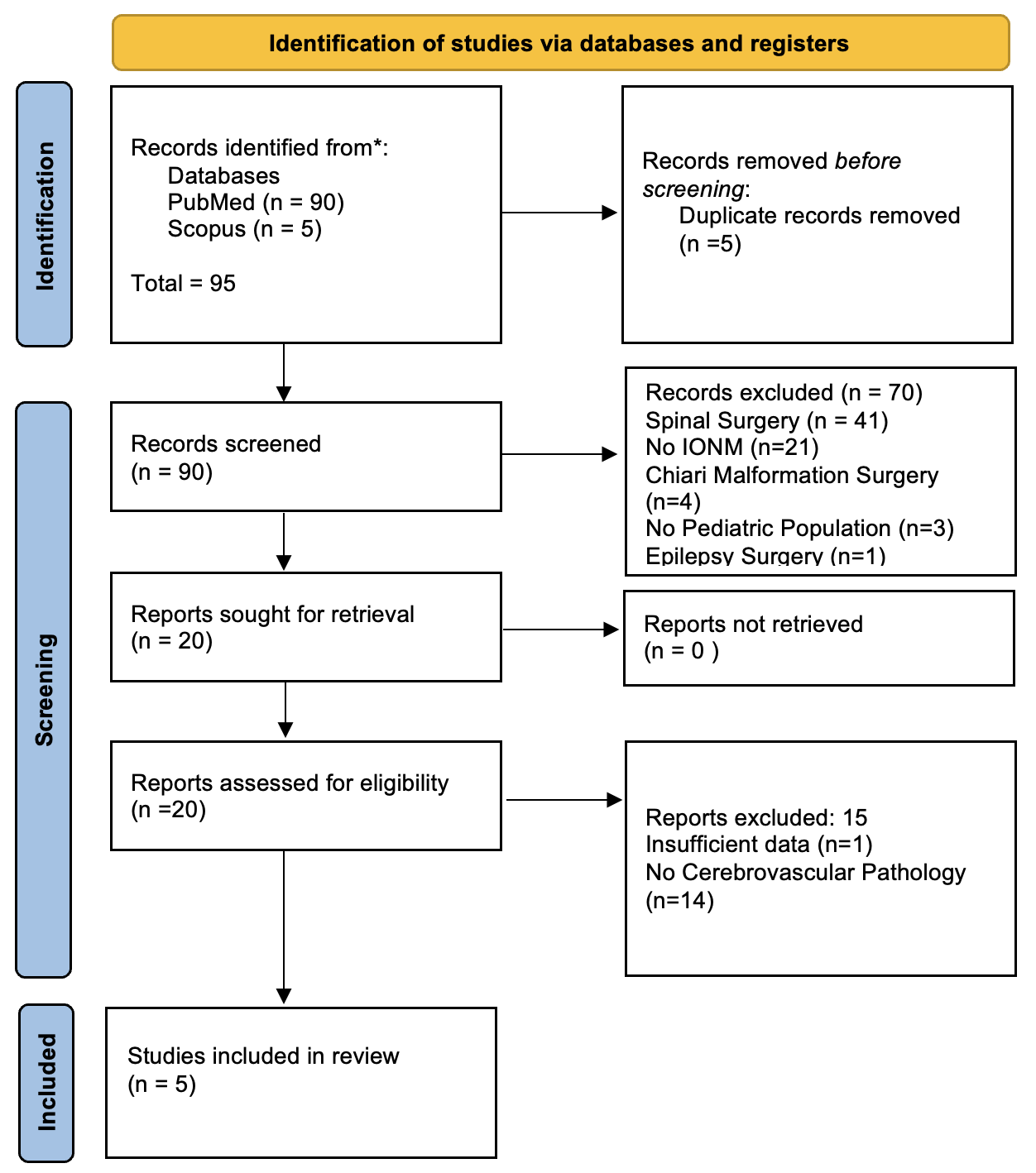

Supplement: Supplementary file 1 — JPEG (190 KB) [file 381_2026_7228_MOESM1_ESM.jpeg]
